# Food-drug interactions: Modelling knowledge and attitude among healthcare professionals at the Ho Teaching Hospital

**DOI:** 10.1371/journal.pone.0323793

**Published:** 2025-05-22

**Authors:** Donatus Wewura Adongo, David Adedia, Augustine Tandoh, Hilda Amekyeh, Killian Asampana Asosega, Charles Kwaku Benneh, Eli Yao Lawluvi, Inemesit Okon Ben, Abigail Hohoayi, Ebenezer Wiafe, Jones Ofori-Amoah, Salifu Nanga, Eric Woode

**Affiliations:** 1 Department of Pharmacology and Toxicology, School of Pharmacy, University of Health and Allied Sciences, Ho, Ghana; 2 Department of Basic Sciences, School of Basic and Biomedical Sciences, University of Health and Allied Sciences, Ho, Ghana; 3 Department of Pharmaceutics, School of Pharmacy, University of Health and Allied Sciences, Ho, Ghana; 4 Department of Mathematics and Statistics, University of Energy and Natural Resources, Sunyani, Ghana; 5 Department of Physician Assistantship, School of Medicine, University of Health and Allied Sciences, Ho, Ghana; 6 Department of Pharmacy Practice, School of Pharmacy, University of Health and Allied Sciences, Ho, Ghana; 7 Discipline of Pharmaceutical Sciences, College of Health Sciences, University of KwaZulu-Natal, Durban, South Africa; 8 Department of Medicine Information and Research, Directorate of Pharmacy, Ho Teaching Hospital, Ho, Ghana; University of Ghana School of Physical and Mathematical Sciences, GHANA

## Abstract

**Background:**

Food-drug interactions (FDIs) are a significant clinical concern, impacting the effectiveness and safety of treatments. Healthcare professionals (HCPs) play a crucial role in minimizing these risks through appropriate patient education. The Ho Teaching Hospital (HTH) in Ghana serves a large patient population with varying medication needs. However, there is limited data on the knowledge and attitudes of HCPs regarding FDIs in this setting.

**Aim:**

This study assessed and modelled the knowledge and attitudes of HCPs towards FDIs at HTH.

**Methodology:**

A cross-sectional survey of 300 HCPs, including medical doctors, pharmacists, nurses, midwives, and dietitians, was conducted using a self-administered questionnaire. Data were analyzed using Kruskal-Wallis tests, logistic regression models and path analysis.

**Results:**

The mean knowledge score on FDIs was 27.52 (SD = 8.71) out of 61, indicating low knowledge. Pharmacists scored the highest, while profession and sex significantly influenced knowledge levels (*p* < 0.05). Only 39% of participants demonstrated high knowledge, and 49.70% exhibited a good attitude towards FDIs. Males were less likely to show good attitudes compared to females (aOR = 0.15; *p* = 0.038). HCPs who attended FDI training (aOR = 1.83; *p* = 0.027) and Christians (aOR = 2.17; *p* = 0.048) displayed more favorable attitudes. The mediation analysis revealed that knowledge of alcohol-drug interactions influences overall attitudes through pathways involving knowledge of drug-food time intervals.

**Conclusion:**

Knowledge of FDIs among HCPs is inadequate, with significant variability across professions. Pharmacists demonstrated the highest knowledge levels, while overall attitudes varied by sex, training, and religious affiliation. These findings highlight the need for targeted educational programs and training for HCPs regarding FDIs.

## Introduction

Food-drug interactions (FDIs) are a significant concern in clinical practice, affecting the effectiveness and safety of pharmacotherapy. These interactions occur when food or drinks affect the absorption, metabolism, or excretion of a drug, potentially leading to reduced positive therapeutic outcomes or increased toxicities [1–3].

The complexity of FDIs arises from the multitude of factors that influence drug pharmacokinetics and pharmacodynamics. These factors include the physicochemical properties of the drug, the nature of the food, the timing of food intake relative to drug administration, and patient-specific variables such as age, genetic profile, and health status [4,5]. FDIs represent an essential and widely under-recognized source of medication errors, which leads to treatment failure, or increased bioavailability, which increases the risk of adverse events and may even precipitate toxicities [1,3,6].

Several studies have highlighted common FDIs, such as the impact of grapefruit juice on the metabolism of certain statins and calcium channel blockers [7–9], and the effect of high-fat meals on the absorption of lipophilic drugs [10,11]. Other well-established clinical examples of FDIs include those between foods containing tyramine and monoamine-oxidase inhibitors (MAOIs); alcoholic beverages or spirits and central nervous system (CNS) drugs; vitamin K, following the ingestion of large quantities of vegetables rich in the vitamin (broccoli, parsley, spinach) and warfarin; as well as those between milk and other dairy products and tetracycline [6,9,12–16]. These interactions can lead to adverse drug reactions or therapeutic failure, underscoring the need for healthcare professionals (HCPs) to possess comprehensive knowledge and a proactive attitude towards identifying and managing these interactions. Patients must be properly informed about these FDIs by HCPs in order for them to know which foods and beverages to avoid when taking medications and when to take them. Therefore, ensuring that HCPs have sufficient knowledge about FDIs is essential [4,17,18].

Previous studies have demonstrated varying levels of awareness and understanding of FDIs among HCPs globally. A study conducted in Ethiopia revealed that HCPs had inadequate knowledge of FDIs [3]. Similar results were observed among HCPs in public sector hospitals in eThekwini, KwaZulu-Natal [4]. In addition to the aforementioned studies, a broader examination of the literature reveals a consistent theme of inadequate knowledge among HCPs globally. Studies among pharmacists in Palestine and Jordan have also revealed unsatisfactory knowledge regarding FDIs [6,19]. Among nurses in Egypt [20] and the United States of America [21], low level of awareness and knowledge regarding FDIs have also been recorded, respectively. A national assessment in Saudi Arabia highlighted a pervasive lack of awareness regarding the potential hazards of FDIs, not only among the general populace but also among HCPs [22]. A similar study conducted in the Eastern region of Saudi Arabia among HCPs also revealed a low level of knowledge about specific FDIs, which pose risks of adverse effects and reduced drug efficacy [23]. Therefore, evidence from multiple studies conducted in various countries consistently highlights a substantial lack of awareness and understanding of FDIs among HCPs. This underscores the necessity for comprehensive educational programs aimed at enhancing the understanding of FDIs among HCPs, which is essential for ensuring patient safety and effective healthcare delivery.

Inadequate knowledge and attitudes toward FDIs among HCPs can significantly compromise patient safety, particularly in the Ghanaian context where the healthcare system is increasingly managing patients with chronic conditions requiring complex medication regimens, coupled with diverse dietary practices [24–26]. Limited awareness of FDIs may lead to inappropriate prescribing, dispensing, or patient counseling, resulting in reduced therapeutic efficacy, adverse drug reactions, and medication errors. These risks are further amplified by the widespread use of herbal remedies and culturally specific dietary practices, which may predispose patients to clinically significant FDIs. Despite the global recognition of FDIs as a critical patient safety concern, there remains a notable lack of empirical research in sub-Saharan Africa, including Ghana—a gap that is especially concerning in light of the rising prevalence of polypharmacy and the common use of indigenous and herbal medicines. This highlights the urgent need to assess the knowledge and attitudes of HCPs toward FDIs within the local healthcare setting.

The Ho Teaching Hospital (HTH), as a major tertiary healthcare facility in the Volta Region, serves a large patient population with varying medication needs. However, there is limited data on the knowledge and attitudes of HCPs regarding FDIs in this setting. This knowledge gap is concerning, as inadequate understanding of FDIs among healthcare providers could compromise patient care and safety.

This study seeks to evaluate and model the knowledge and attitudes of HCPs at HTH regarding FDIs through the use of a self-administered questionnaire. Additionally, it examines how knowledge of alcohol-drug interactions (ADIs) influences the relationship between FDI knowledge, drug-to-food time intervals (DFTIs), and HCP attitudes toward FDIs. By understanding the current level of awareness, identifying knowledge gaps, and exploring factors that influence HCPs’ attitudes toward FDIs, this research will provide valuable insights for developing targeted educational interventions and improving clinical practice guidelines. The findings will contribute to the broader literature on FDIs while addressing a critical aspect of medication safety in the Ghanaian healthcare system.

## Methodology

### Study design and setting

This was a descriptive cross-sectional study conducted to assess the knowledge and attitude of HCPs at HTH towards FDIs. The study was conducted among medical doctors, pharmacists, nurses, midwives and dietitians from September to November 2023. The data collection period coincided with peak staff presence across departments, allowing for sufficient diversity and representation of HCPs to capture variation in knowledge and attitudes toward FDIs. HTH is the main referral health facility in the Volta Region and receives patients from all the peripheral facilities in the Volta and Oti regions as well as parts of the Eastern region of Ghana. The hospital is also patronized by patients from neighboring countries like Togo and Benin. The clinical departments include surgical, internal medicine, obstetrics and gynecology, child health, and public health. The hospital also runs special clinics like; eye clinic, sickle cell clinic, anti-retroviral clinic, infertility clinic, and dental clinic. The staff strength of the hospital at the time of this study was 1545 comprising 90 medical doctors, 24 pharmacists, 574 nurses (enrolled nurses, staff nurses and nursing officers), 124 auxiliary nurses, 164 midwives (all ranks), 4 dietitians, and other hospital professionals.

### Study participants

A total of 980 HCPs (medical doctors, pharmacists, nurses, midwives, and dietitians) formed the target population for this study. Due to the low number of pharmacists, all consenting pharmacists working in the hospital were recruited to participate in the study. In addition, the low number of dietitians at the facility warranted the need to include in this study, consenting interns and non-core hospital staff dietitians (specifically, dietitians from the University of Health and Allied Sciences) who render services at the dietetics unit of the hospital. On-duty medical doctors, nurses, and midwives were conveniently selected from the outpatient and inpatient wards of the hospital. Other HCPs, such as physiotherapists and radiologists, were excluded from this study because they are not actively involved in prescribing, dispensing or administering medications to patients.

Medical doctor: An HCP licensed to diagnose, treat, and prevent diseases and medical conditions. In the context of this study, medical doctors included general practitioners and specialists.

Pharmacist: A licensed HCP specialized in the safe and effective use of medications.

Nurse: An HCP responsible for patient care, which involves administering medications, monitoring patient health, and providing support and education to patients and their families.

Midwife: A specialized HCP who provides care to women during pregnancy, childbirth, and the postpartum period.

Dietitian: Expert in food and nutrition, responsible for developing and implementing personalized nutrition plans to promote health or manage medical conditions.

### Sample size

The sample size for this study was calculated using Yamane’s formula as previously described [27].


$$\begin{array}{c} \text{n }=\frac{N}{1+N{\left(e\right)}^{2}} \end{array}$$

Given the target population (N) of 980, the degree of desired accuracy (e) was set at 0.05. Using a 95% confidence level, a sample size (n) of 298 was determined with a non-response rate of 5%. However, a sample of 300 participants was considered.

A convenience sampling method was used in this study, and comprised HCPs from all categories of staff, including those working night shifts, as long as they met the inclusion criteria. This comprehensive approach aimed to ensure that a diverse range of HCPs were represented in the study, reducing the likelihood of selection bias and enhancing the robustness of our findings. Participants were recruited once they fell within the inclusion criteria. A systematic approach to minimize potential non-response was implemented. Specifically, we utilized several strategies to encourage participation, such as sending follow-up reminders and approaching participants at times that would cause minimal disruption to their duties.

### Data collection tool, and handling

An anonymous self-administered questionnaire was used to collect data for the study. The questionnaire was adapted from previously validated tools used in similar studies [4,19,21], with modifications made to reflect the local healthcare context and study objectives. The questionnaire contained 40 close-ended questions and one open-ended question. The questionnaire gathered information on sociodemographic characteristics including sex, age, level of education, occupation, years of work experience, and attendance of training on FDIs. The knowledge questions centered on general knowledge of FDIs (total score of 4, Cronbach’s alpha = 0.64) and knowledge of interactions between food and some specific drugs (total score of 39, Cronbach’s alpha = 0.84), knowledge of food-to-drug-time intervals (total score of 11, Cronbach’s alpha = 0.63), knowledge of alcohol-drug interactions (ADIs) (total score 7, Cronbach’s alpha = 0.85), and the attitude of HCPs towards FDIs (4 items, total score of 8, Cronbach’s alpha = 0.63) were reliable. The individual components of the knowledge questions, as well as the overall knowledge items, were reliable (Cronbach’s alpha = 0.86). The objective of the open-ended question was to get suggestions on how knowledge of FDIs can be improved. A score of 1 was assigned to each correct answer and 0 to a wrong answer or ‘I don’t know’ on questions regarding knowledge of FDIs. The questionnaire was piloted among 10 selected HCPs working at HTH to assess clarity, relevance, and comprehension. Based on the feedback, minor adjustments were made to the wording and structure of some items to enhance clarity and ensure consistency, thereby improving the overall reliability of the instrument. These 10 people were excluded from the study. The reliability test was conducted using the data collected from the main study and not from the literature or the 10 piloted participants.

An average knowledge score of 27.52 was obtained. Hence, participants who scored at most 27.52 were assigned low knowledge while scores of more than 27.52 were assigned high knowledge [4]. Participants who responded accurately to more than three-quarters of all the attitude questions were adjudged as having a good overall attitude.

All collected data were examined for completeness and consistency during data management, storage, and analysis. Response completeness was high, as careful monitoring and guidance during data collection minimized the likelihood of incomplete responses, thereby reducing any potential impact on the study outcomes. Additionally, data were double-checked during entry to minimize errors and maintain consistency. The collected information was kept anonymous through the use of non-identifying numbers. Electronic data and completed surveys were kept safe in a closed file cabinet that only the lead investigator and academic supervisor had access to. Data on computers were password-protected.

### Ethical consideration

Ethical approval was sought from the Research Ethics Committee of the University of Health and Allied Sciences (approval number: REC A. 1 [25] 23–24). Permission was sought from the administration of the Ho Teaching Hospital before the participants were engaged in data collection. Participants in this study were informed about the research’s purpose, procedures, and potential benefits. Participation was entirely voluntary, and written informed consent was obtained from all participants before data collection. Participants were assured of the confidentiality and anonymity of their responses, and they were given the right to withdraw from the study at any stage without any consequences. All data collected were treated with strict confidentiality by identifying participants with number labels instead of names, and the data were used solely for this research.

### Statistical analysis

Using an alpha of 5%, Kruskal Wallis with Dunn’s multiple comparison tests were employed to compare scores across more than two groups, while the Mann-Whitney U test was used to compare scores across two groups. The non-parametric statistical methods were used for the comparisons of scores because the assessment of the distributions of knowledge and attitude scores showed that they deviated slightly from normality.

### The logistic regression model

Logistic regression models were used to predict participants’ knowledge and attitude levels. The binary logistic regression model is a flexible and robust statistical tool used to implement binary classification for a categorical (dichotomous) target variable [28]. The logistic model estimates and describes the relationship between a binary target variable (such as present/absent of a condition, success/failure or yes/no) and a set of categorical and or quantitative covariates. In contrast to the linear regression model which models a continuous outcome, the logistic model does not model the categorical variable Y, but its probability of occurrence using the logit transformation [29] in the form:


$$\begin{array}{c} LogitP(x)=Log\left(\frac{p}{1-p}\right)=\;\beta_{0}+\beta_{1}X_{1}+\beta_{2}X_{2}+\;...+\;\beta_{k}X_{k} \end{array}$$ (1)

Where $\beta_{1},\;\beta_{2},\;\cdots ,\;\beta_{k}$ are coefficients of covariates $X_{1},\;X_{2},\;\cdots ,\;X_{k}$ and p is the probability of the condition. The probability of any given observation is expressed as


$$P(x)=\frac{e^{\left(\beta_{0}+\beta_{1}X_{1}+\beta_{2}X_{2}+\;..\;.+\;\beta_{k}X_{k}\right)}}{1+\;e^{\left(\beta_{0}+\beta_{1}X_{1}+\beta_{2}X_{2}+\;..\;.+\;\beta_{k}X_{k}\right)}}$$ (2)

The model parameters $\beta_{1},\;\beta_{2},\;\cdots ,\;\beta_{k}$ are estimated using the maximum likelihood estimation method through the Newton- Raphson iterative algorithm [30,31].

### Test of significance and model evaluation

Hosmer-Lemeshow and likelihood ratio tests as well as Nagelkerke and Cox and Snell metrics were used to assess the logistic regression models as previously employed by Adongo and colleagues [32]. The significance of model parameters linked to covariates in the model was assessed through the Wald test which is the square of the ratio of the covariate’s coefficient $\beta_{i}$ to its standard error and follows a standard normal distribution [33].

The overall goodness-of-fit of the fitted model to assess how well the model fits the data is determined by the Hosmer-Lemeshow test [34]. The Hosmer-Lemeshow procedure tests the null hypothesis, $H_{0}\;:\;$the model adequately fits the data against $H_{1}\;:\;$the model does not fit the data. The desirable outcome of the Chi-square test statistic of non-significance usually verifies that the model predictions are not significantly different from the observed data [35].

The goodness-of-fit of the logistic model can also be assessed based on the likelihood ratio test (LRT) which is the ratio of the maximized value of the likelihood function for the simpler model (LM_N_) over the maximized value of the likelihood function for the full model (LM_F_) [36–38]. The likelihood-ratio test statistic equals


$$-2Log\left(\frac{{LM}_{N}}{{LM}_{F}}\right)=-2(Log{\;LM}_{N}-Log{LM}_{F})$$ (3)

This log transformation of the likelihood functions is an asymptotically chi-squared statistic with degrees of freedom equal to the difference in the number of parameters between the full and simple models. The simple model ($M_{N}$) is usually nested in the full model ($M_{F}$) and a resulting significant LRT (p<0.05) shows a significant contribution of covariates added to the simple model [36].

In modelling knowledge and attitude of HCPs towards FDIs, confounding variables such as education, age, sex, and years of professional experience were controlled for using logistic regression models. The assumptions assessed for the logistic regression model include independence of the observations, a dichotomous response variable, no multicollinearity, no extreme values or outliers, a sufficiently large sample size, and linearity.

### Mediation analysis

Mediation analysis was undertaken to assess the mediation effects of knowledge of ADIs in explaining the relationship between the attitude of participants and their knowledge of FDIs as well as knowledge of drug-to-food time intervals. Mediation analysis helps to determine the direct and indirect effects of exogenous variables [39,40]. Comparative Fit Index (CFI), Tucker-Lewis Index (TLI), Root Mean Square Error of Approximation (RMSEA) and Standardized Root Mean Square Residual (SRMR) were used to assess the fitness of the mediation models [39,40].

The mediation models were fitted using the maximum likelihood estimator (MLE) in Eq (4), where the implied covariance matrix, sample covariance matrix, number of observed variables and trace were Σ(θ), *S, p and tr*, respectively.


$$\begin{array}{c} \text{FML }=\text{ log }\Sigma (\theta )\;-\text{ log S }+\text{ tr}[{\text{S}\Sigma (\theta )}^{-1}]-\text{ p } \end{array}$$ (4)

The assessment of the data regarding multivariate normality assumption showed a slight deviation. The MLE does well with multivariate normal data or data which slightly deviate from multivariate normality [39,40].

## Results

### Demographic characteristics of HCPs

From Table 1, most of the participants were females (62.00%), aged 25–30 (63.30%), Christians (86.00%), and had less than five years of working experience (78.00%). More than 50% of the participants were nurses (54.30%), and had a Bachelor’s degree (50.30%), while less than 50.00% attended a training at which they were informed about FDIs (42.70%).

**Table 1:** Demographic characteristics of HCPs.

Characteristics	N	%
**Sex**		
Male	114	38.00
Female	186	62.00
**Age (years)**		
<25	37	12.30
25-30	190	63.30
>30	73	24.30
**Religion**		
Christian	258	86.00
Non-Christian	42	14.00
**Profession**		
Medical Doctor	59	19.70
Pharmacist	15	5.00
Nurse	163	54.30
Dietitian	16	5.30
Midwife	47	15.70
**Level of education**		
Certificate/Diploma	149	49.70
Bachelor’s/Master’s	151	50.30
**Years of work experience**		
0-4 years	234	78.00
5-9 years	37	12.30
10-14 years	29	9.70
**Attended training on FDIs**		
No	172	57.30
Yes	128	42.70

### Overall knowledge and attitude

Among the 300 participants, 39.00% had high knowledge while 49.70% had good attitude towards FDIs (Table 2). Out of a total knowledge score of 61, the mean (SD) of overall knowledge was 27.52 (8.71).

**Table 2:** Ranked correct responses to the knowledge of FDIs, drug-to-food time interval and ADIs.

Items	N	%
**General knowledge of FDIs**		
Some foods can interfere with the effectiveness of drugs in the body	295	98.30
Some foods can increase or decrease the action of a drug	293	97.70
Some drinks can interfere with the effectiveness of drugs in the body	291	97.00
Some drugs can alter the nutritional status of a patient	275	91.70
**Knowledge of FDIs**		
Antibiotics with citrus juice	246	82.00
Theophylline with coffee	232	77.30
Tetracycline and fluoroquinolones with milk	200	66.70
Diazepam with energy drinks	190	63.30
Tetracycline and fluoroquinolones with dairy products	178	59.30
Levodopa with egg	167	55.70
Diazepam with coffee	164	54.70
Spironolactone with spinach	139	46.30
Atorvastatin with grape fruit juice	135	45.00
Warfarin with green leafy vegetables	131	43.70
MAOIs with fermented foods	125	41.7
Levodopa with beef	124	41.30
Warfarin with spinach	123	41.00
Spironolactone with broccoli	123	41.00
Levothyroxine with cabbage	122	40.70
Warfarin with broccoli	119	39.70
Theophylline with chocolate	116	38.70
Diltiazem with grapefruit juice	114	38.00
Spironolactone with green leafy vegetables	111	37.00
MAOIs with wine	97	32.30
Theophylline with tea	81	27.00
Tetracycline and fluoroquinolones with iron-rich food	43	14.30
**Knowledge of drug-to-food time interval**		
Esomeprazole/omeprazole with food	176	58.70
Taking zidovudine without food	112	37.30
Isoniazid with food	103	34.30
Griseofulvin and albendazole with high-fat foods	102	34.00
Metformin with food	101	33.70
Taking ACE inhibitors on an empty stomach	93	31.00
Lopinavir/ritonavir with food	86	28.70
Taking didanosine and indinavir on an empty stomach	82	27.30
Taking propranolol on an empty stomach	82	27.30
Glipizide with food	82	27.30
NSAIDs and steroids with food	55	18.30
**Knowledge of ADIs**		
Diazepam	263	87.7
Metronidazole	254	84.7
Metformin	238	79.3
Antihistamines	230	76.7
Co-trimoxazole	224	74.7
Methotrexate	217	72.3
Isoniazid	200	66.7
**Overall knowledge**		
Low	183	61.0
High	117	39.0
**Overall attitude**		
Poor	151	50.3
Good	149	49.7

### General knowledge of FDIs

The general knowledge questions had a mean (SD) score of 3.85 (0.53). Concerning the individual items (Table 2), most of the participants knew that some foods can interfere with the effectiveness of drugs in the body (98.30%). The majority of participants agreed that some foods can increase or decrease the action of a drug (97.70%), some drinks can interfere with the effectiveness of drugs (97.00%) and lastly, some drugs can alter the nutritional status of a patient (91.70%).

### Knowledge of common FDIs

Out of a total score of 39, the knowledge on specific FDIs reported a mean (SD) score of 14.67 (6.60). As shown in Table 2, most of the participants knew avoidance of antibiotics with citrus juice (82.00%), followed by avoidance of theophylline with coffee (77.30%), avoidance of tetracycline and fluoroquinolones with milk (66.70%), avoidance of diazepam with energy drinks (63.30%), avoidance of tetracycline and fluoroquinolones with dairy products (59.30%), avoidance of levodopa with egg (55.70%), and avoidance of diazepam with coffee (54.70%). Less than 50.00% of the participants had knowledge of the remaining items. These include avoidance of spironolactone with spinach (46.30%), atorvastatin with grapefruit juice (45.00%), avoidance of warfarin with green leafy vegetables (43.70%), avoidance of MAOIs with fermented foods (41.70%), and levothyroxine with cabbage (40.70%).

### Knowledge of drug-to-food time interval

Out of a total score of 11, the mean (SD) knowledge of drug-to-food time interval was 3.58 (2.32). Regarding the individual items in Table 2, 58.70% of the participants showed good knowledge of esomeprazole/omeprazole with food. Less than 50.00% of the HCPs provided correct responses regarding taking zidovudine (37.30%), isoniazid (34.30%), griseofulvin and albendazole (34.00%), metformin (33.70%), and taking angiotensin-converting enzyme (ACE) inhibitors (31.00%) with food.

### Knowledge of ADIs

Out of a total score of 7, knowledge of ADIs reported a mean (SD) of 5.42 (2.09). Among the individual items in Table 2, 87.70% knew the avoidance of diazepam with alcohol. Others knew the avoidance of metronidazole (84.70%), metformin (79.30%), and antihistamines (76.70%), co-trimoxazole (74.70%), methotrexate (72.30%) and isoniazid (66.70%) with alcohol.

### Demographic variations in knowledge and attitude

As shown in Table 3, respondents with Bachelor’s or Master’s degree reported a higher knowledge score of FDIs than certificate or diploma holders (*p* = 0.049). The general knowledge scores of participants did not vary by any demographic feature. The Knowledge of FDI scores for pharmacists was significantly highest compared to medical doctors (*p* = 0.002), nurses (*p* = 0.001), dietitians (*p* = 0.006) and midwives (*p* < 0.001). Participants with previous FDI training reported higher scores than those without (*p* = 0.040).

**Table 3:** Demographic variations in knowledge and attitude of participants.

Characteristics	Attitude towards FDIs	General knowledge (KG1)	Knowledge of FDIs (KG-FDIs)	Knowledge of drug-to-food time interval (KG-DFTI)	Knowledge of ADIs (KG-ADIs)	Overall knowledge (KGT)
**Sex**	Mean ± SD	Mean ± SD	Mean ± SD	Mean ± SD	Mean ± SD	Mean ± SD
Male	5.17^b ^± 1.56	3.84 ± 0.54	14.31 ± 6.65	3.39 ± 2.36	5.00^b ^± 2.25	26.54 ± 9.13
Female	5.54^a ^± 1.67	3.85 ± 0.52	14.90 ± 6.58	3.70 ± 2.30	5.68 ^a ^± 1.95	28.12 ± 8.41
***p*-value**	**0.032**	0.979	0.439	0.246	**0.002**	0.061
**Religion**						
Christian	5.47 ± 1.58	3.85 ± 0.48	14.62 ± 6.44	3.71^a^ ± 2.32	5.48 ± 1.98	27.66 ± 8.55
Non-Christian	4.93 ± 1.92	3.81 ± 0.77	14.98 ± 7.59	2.79^b^ ± 2.21	5.07 ± 2.64	26.64 ± 9.71
***p*-value**	0.083	0.479	0.942	**0.013**	0.855	0.366
**Profession**						
Medical Doctor	4.42^b^ ± 1.48	3.86 ± 0.60	14.51^b^ ± 7.36	3.37 ^b^ ± 2.37	4.34^b ^± 2.54	26.08^b ^± 10.26
Pharmacist	5.73^a ^± 1.33	3.67 ± 1.05	20.93 ^a ^± 5.20	6.53^a ^± 2.26	6.40^a ^± 1.12	37.53^a ^± 6.81
Nurse	5.59^a ^± 1.60	3.82 ± 0.51	14.79^b^ ± 6.47	3.38^b^ ± 2.1	5.63^a ^± 1.94	27.62^b ^± 8.09
Dietitian	5.44^a ^± 1.71	4.00 ± 0.00	14.12^b^ ± 7.31	2.69^b^ ± 1.3	5.25^ab ^± 2.41	26.06^b ^± 9.21
Midwife	5.83^a ^± 1.61	3.91 ± 0.28	12.66^b^ ± 4.99	3.89^b^ ± 2.63	5.81^a ^± 1.60	26.28^b ^± 7.09
***p*-value**	**<0.001**	0.344	**<0.001**	**<0.001**	**<0.001**	**<0.001**
**Education**						
Certificate/Diploma	5.77^a ^± 1.47	3.82 ± 0.57	13.97^b ^± 6.10	3.53 ± 2.16	5.86 ^a ^± 1.70	27.17 ± 7.83
Bachelor’s/Master’s	5.03^b ^± 1.71	3.87 ± 0.48	15.37^a ^± 7.01	3.63 ± 2.47	4.99^b ^± 2.33	27.86 ± 9.51
***p*-value**	**<0.001**	0.256	**0.049**	0.986	**<0.001**	0.593
**Attended training on FDIs**						
No	5.17^b ^± 1.71	3.84 ± 0.52	14.12 ^b ^± 6.53	3.58 ± 2.14	5.19 ± 2.25	26.72 ± 8.37
Yes	5.70^a ^± 1.49	3.85 ± 0.53	15.42 ^a ^± 6.65	3.59 ± 2.55	5.73 ± 1.81	28.59 ± 9.07
***p*-value**	**0.015**	0.803	**0.044**	0.534	0.064	0.093
**Age (years)**						
<25	5.30 ± 1.39	3.89 ± 0.66	12.54 ± 5.70	3.19 ± 2.50	5.11 ± 2.57	24.73 ± 8.76
25-30	5.44 ± 1.62	3.84 ± 0.53	14.88 ± 6.34	3.72 ± 2.29	5.51 ± 1.92	27.94 ± 8.49
>30	5.33 ± 1.80	3.85 ± 0.43	15.21 ± 7.51	3.42 ± 2.30	5.36 ± 2.24	27.84 ± 9.10
Total	5.40 ± 1.64	3.85 ± 0.53	14.67 ± 6.60	3.58 ± 2.32	5.42 ± 2.09	27.52 ± 8.71
***p*-value**	0.595	0.268	0.053	0.229	0.991	0.054

SD: standard deviation.

Pharmacists reported the highest knowledge of drug-to-food time interval scores compared to the other HCPs (*p* < 0.001) while Christians reported higher scores of knowledge of drug-to-food time intervals than non-Christians (*p* = 0.013).

Medical doctors reported the lowest score of knowledge on ADIs compared with pharmacists (*p* = 0.005), nurses (*p* = 0.002) and midwives (*p* = 0.006). Scores of knowledge on ADIs were significantly higher among females than males (*p* = 0.002), and higher in certificate/diploma holders than Bachelor’s or Master’s holders (*p* < 0.001). Pharmacists significantly reported the highest total scores of knowledge than the other HCPs (*p* < 0.001).

Regarding attitude scores, female participants had better attitude scores than males (*p* = 0.032), participants who attended an FDI training had better attitude scores than those who did not (*p *= 0.015), participants with certificate or diploma education had better attitude scores than those with Bachelor’s/Master’s degree(s) (*p* < 0.001), and medical doctors reported lowest attitude scores compared to pharmacists (*p* = 0.023), nurses (*p* < 0.001), dietitians (*p* = 0.048) and midwives (*p *< 0.001).

### Predictors of overall knowledge

The logistic regression model was accurately fitted with a non-significant Hosmer-Lemeshow test (*p* = 0.721), a significant likelihood ratio test (Chi-square statistic = 38.62, *p* < 0.001), Nagelkerke metric of 0.16 and Cox and Snell metric of 0.12 (Table 4). The multiple logistic regression model for overall knowledge showed that males were less likely to have high overall knowledge as compared to females (aOR = 0.50; 95% CI: 0.27, 0.91; *p* = 0.026). Dietitians (aOR=0.02; 95% CI: 0.00, 0.16; *p* = 0.001), medical doctors (aOR = 0.05; 95% CI: 0.00, 0.29; *p* = 0.006), midwives (aOR = 0.02; 95% CI: 0.00, 0.14; *p* = 0.001) and nurses (aOR = 0.04; 95% CI: 0.00, 0.25; *p* = 0.004) were less likely to have high overall knowledge than pharmacists.

**Table 4:** Predictors of overall knowledge (model 1).

Predictors	aOR (95% CI)	*p*-value
(Intercept)	8.84 (1.14, 190.04)	0.069
Sex [Male]	**0.5 (0.27, 0.91)**	**0.026**
Sex [Ref = Female]		
Religion [Christian]	1.08 (0.51, 2.34)	0.844
Religion [Ref = Non-Christian]		
Profession [Dietitian]	**0.02 (0.00, 0.16)**	**0.001**
Profession [Medical Doctor]	**0.05 (0.00, 0.29)**	**0.006**
Profession [Midwife]	**0.02 (0.00, 0.14)**	**0.001**
Profession [Nurse]	**0.04 (0.00, 0.25)**	**0.004**
Profession [Ref = Pharmacist]		
Education level [Certificate/Diploma]	0.67 (0.36, 1.23)	0.194
Education level [Ref = BSc/Masters]		
Years of experience [10–14 years]	0.64 (0.23, 1.66)	0.378
Years of experience [5–9 years]	1.44 (0.65, 3.19)	0.367
Year of experience [Ref = 0–4 years]		
Attended training on FDIs [Yes]	1.56 (0.93, 2.65)	0.095
Attended training on FDIs [Ref = No]		
Age [>30 years]	1.83 (0.68, 5.31)	0.243
Age [25–30 years]	2.31 (0.99, 5.99)	0.066
Age [Ref= < 25 years]		

### Predictors of overall attitude

The logistic regression model for overall attitude was accurately fitted with a non-significant Hosmer-Lemeshow test (*p* = 0.180), a significant likelihood ratio test (Chi-square statistic = 57.92, *p* < 0.001), Nagelkerke metric of 0.23 and Cox and Snell metric of 0.18 (Table 5). Generally, males (aOR = 0.15; 95% CI: 0.02, 0.78; *p* = 0.038) were less likely to report good attitudes compared with females. However, sex and age have interaction effects on good attitudes, such that males aged between 25 and 30 years (aOR = 10.10; 95% CI: 2.24, 72.95; *p* = 0.007) and males aged above 30 years (aOR = 8.22; 95% CI: 1.53, 66.07; *p *= 0.023) were more likely to have good attitudes than males below 25 years. Participants who had attended training on FDIs (aOR = 1.83; 95% CI: 1.08, 3.13; *p* = 0.027) and Christians (aOR = 2.17; 95% CI: 1.02, 4.78; *p* = 0.048) are more likely to show good attitude than those without FDI attendance and non-Christians, respectively. However, medical doctors (aOR = 0.25; 95% CI: 0.07, 0.89; *p *= 0.033) are less likely to show good attitudes compared to pharmacists.

**Table 5:** Predictors of overall attitude (Model 2).

Predictors	aOR (95% CI)	p-value
Intercept	0.26 (0.05, 1.29)	0.099
Sex [Male]	**0.15 (0.02, 0.78)**	**0.038**
Sex [Ref = Female]		
Religion [Christian]	**2.17 (1.02, 4.78)**	**0.048**
Religion [Ref = Non-Christian]		
Profession [Dietitian]	2.04 (0.43, 9.83)	0.369
Profession [Medical Doctor]	**0.25 (0.07, 0.89)**	**0.033**
Profession [Midwife]	1.21 (0.33, 4.46)	0.769
Profession [Nurse]	1.55 (0.47, 5.08)	0.464
Profession [Ref = Pharmacist]		
Education level [Certificate/Diploma]	1.58 (0.86, 2.91)	0.139
Education level [Ref = BSc/Masters]		
Year of experience [10–14 years]	2.12 (0.84, 5.70)	0.119
Year of experience [5–9 years]	0.79 (0.35, 1.80)	0.573
Year of experience [Ref = 0–4 years]		
Attended training on FDIs [Yes]	**1.83 (1.08, 3.13)**	**0.027**
Attended training on FDIs [Ref = No]		
Sex [Female] × Age [>30 years]	0.83 (0.26, 2.55)	0.740
Sex [Male] × Age [>30 years]	**8.22 (1.53, 66.07)**	**0.023**
Sex [Female]: Age [25–30 years]	1.13 (0.42, 2.97)	0.798
Sex [Male]: Age [25–30 years]	**10.10 (2.24, 72.95)**	**0.007**
Age [Ref= < 25 years]		

### Mediation analysis

The path mediation models (Model 3 and Model 4) were accurately fitted with CFI and TLI values of 1.00 while RMSEA and SRMR values of 0.00, respectively.

**Fig 1:**
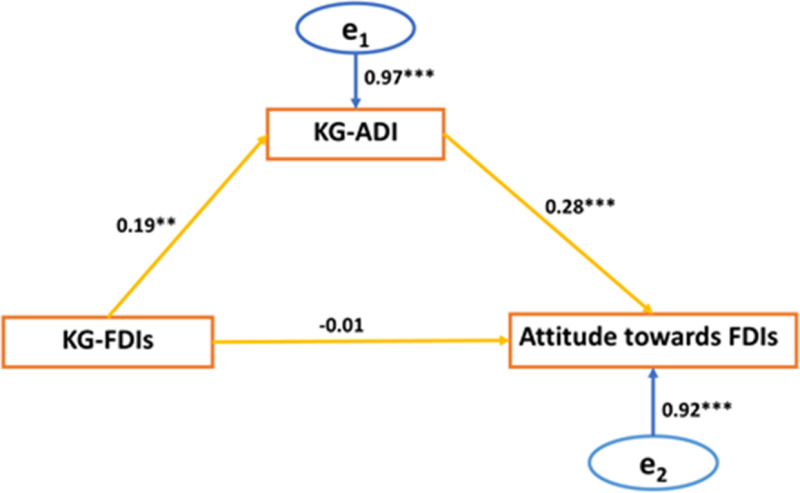
A mediation analysis of knowledge of ADIs (KG-ADIs) on the relationship between knowledge of FDIs (KG-FDIs) and attitude towards FDIs (Model 3).

Knowledge of ADIs mediated the relationship between knowledge of FDIs and attitude (Fig 1). Although the knowledge of FDIs of participants did not directly influence their attitude, it indirectly influenced their attitude. This implies that those with high knowledge of FDIs will have a higher attitude if they have high knowledge of ADIs.

**Fig 2:**
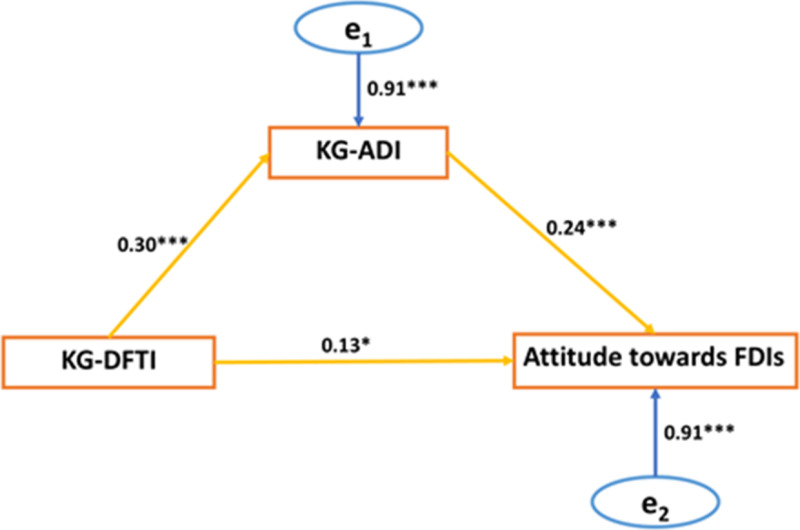
A mediation analysis of knowledge of ADIs (KG-ADIs) on the relationship between knowledge of drug-to-food time interval (KG-DFTI) and attitude towards FDIs (Model 4). Attitude towards FDIs, knowledge of FDIs (KG-FDIs), knowledge of drug-to-food time interval (KG-DFTI), knowledge of ADIs (KG-ADIs); e_1_ and e_2_ are errors associated with knowledge of ADIs and attitude towards FDIs, respectively in both path models.

Knowledge of ADIs mediated the relationship between Knowledge of drug-to-food time interval and attitude (Fig 2). Knowledge of drug-to-food time intervals directly and indirectly influenced participants’ attitudes. This shows that, although participants with high knowledge of drug-to-food time intervals have better attitudes, their attitudes will be improved with a high knowledge of ADIs.

## Discussion

Understanding FDIs is crucial for optimizing patient care and the effectiveness of pharmacotherapy. This study aimed to assess the knowledge and attitudes of HCPs at the Ho Teaching Hospital regarding FDIs. The present findings contribute to the limited literature available in Ghana about HCPs’ knowledge of FDIs and allow for future comparative work and research worldwide. The recruitment of a varied pool of HCPs from different fields including medical doctors, pharmacists, nurses, midwives and dietitians offers a broad assessment of the level of understanding on the subject of FDIs in the healthcare setting. The results could provide insights into current educational gaps and potential areas for improvement in clinical practice.

The findings of this study revealed a very high general knowledge of FDIs (96.25%) among HCPs. Concerning the individual items, most of the participants were aware that some foods and drinks can interfere with the effectiveness of drugs in the body. In addition, the majority of HCPs were aware that some foods can increase or decrease the action of a drug, and that some drugs can alter the nutritional status of a patient. These findings are in agreement with other studies, which also reported high general knowledge on FDIs among HCPs [3,4,23]. The HCPs’ extensive general knowledge of FDIs may stem from their training, which included information on how certain foods and drinks potentially interact with medications in the body.

Although participants had high general knowledge of FDIs, their overall knowledge of FDIs was found to be low (45.11%), which is consistent with studies done in different countries worldwide [3,4,6,19,21,23,41]. The knowledge score in this study was however lower than the findings reported from studies in South Africa (49.26%) [4], Ethiopia (48.40%), Palestine (61.70%) [6] and Jordan (60.00%) [19] among HCPs. The fact that only pharmacists participated in the studies conducted in Jordan and Palestine may account for their higher knowledge of FDIs when compared to our study, and those conducted in South Africa and Ethiopia.

The disparity between the high overall knowledge of FDIs and the lower specific knowledge reveals a critical gap with significant implications for patient care in Ghana. This gap in detailed knowledge could negatively impact patient care, potentially leading to medication errors, reduced treatment effectiveness, and adverse events, especially for patients with chronic conditions. Addressing this issue through targeted educational interventions is essential to improve medication management and ensure patient safety in Ghana’s healthcare system.

The HCPs exhibited varied knowledge in identifying specific food items that can interact with drugs. For instance, about 77.30% of the participants were able to recognize the interaction between theophylline and coffee, which is quite similar to a study conducted in Ethiopia (78.09%) among HCPs [3] but less than the study in South Africa [4]. In contrast, participants in this study poorly identified chocolate and tea as foods that can interact with theophylline, which is also similar to an earlier study [4]. Theophylline is a derivative of xanthine which is equally present in these foods, hence large consumption increases the plasma level of the drug increasing side effects like nausea, vomiting and irritability [5].

Dairy products contain multivalent ions such as calcium and magnesium that have been shown to chelate many medicines, including tetracycline and fluoroquinolones, forming non-absorbable complexes. The formation of these complexes would decrease the absorption of the aforementioned medications [42,43]. Similar to a study conducted in Ethiopia [3], about two-thirds of HCPs in this study were aware of interactions between tetracycline and fluoroquinolones with milk, whereas less than 30.00% of HCPs knew about these drugs interactions with dairy products and iron-rich food. Our study however showed a higher awareness of the interaction between tetracycline/fluoroquinolones and dairy products. In addition, this study showed a higher knowledge regarding the interaction between tetracycline/fluoroquinolones and milk than the study by Osuala and colleagues [4]. Studies conducted among pharmacists in Jordan (87.30%) [19] and in Palestine (94.21%) [6] reflect comparatively higher knowledge about tetracycline interaction with milk and dairy products. This discrepancy might have resulted as the latter studies included only pharmacists.

Another significant finding from this study was the interaction between spironolactone and potassium-rich foods. Spironolactone is a potassium-sparing diuretic with hyperkalemia as its possible side effect. Consuming potassium-rich foods with spironolactone potentially increases the risks of hyperkalemia manifesting clinically as cardiac arrhythmia and muscle paralysis [44]. In a study conducted in Jordan, only 53.30% of the participants were aware of the interaction between spironolactone and potassium-rich foods [19]. Meanwhile, the current study showed that less than 50.00% of the HCPs knew that spironolactone can interact with spinach, broccoli and other green leafy vegetables. This is quite worrying as the abovementioned interaction can potentially cause hyperkalemia.

The effects of anticoagulants such as warfarin are affected by foods high in vitamin K, such as spinach, broccoli, and other green leafy vegetables, as they may promote the synthesis of clotting factors, which lessens the drug’s therapeutic effects [45,46]. In this study, most of the HCPs failed to identify spinach, broccoli and other green leafy vegetables as foods that can interact with the therapeutic effects of warfarin. These findings were consistent with the previous findings [3]. Similarly, a study by Osuala and colleagues also reported that, except for spinach, the HCPs poorly identified other foods that can interact with warfarin [4].

When combined with MAOIs, tyramine-rich foods including cheese, wine, beer, and other fermented foods have the potential to cause a hypertensive crisis. This is brought on by a decrease in the breakdown of tyramine, which results in a build-up in the circulation where it is taken up by noradrenergic neurons, leading to a hypertensive crisis [23,46]. The findings from this study revealed that the majority of the participants had low knowledge of the interaction between MAOIs and tyramine-containing foods, which is in agreement with the findings reported in studies conducted in South Africa and Ethiopia [3,4]. This could be a result of the replacement of MAOIs by newer generation antidepressants. However, the findings from this study disagrees with studies conducted in Palestine [6] and Jordan [19] regarding HCPs’ knowledge of interactions between MAOIs and tyramine-rich foods. HCPs’ awareness about such interactions and counselling patients about them could prevent life-threating events.

Concerning grapefruit juice and drug interactions, four drugs were assessed: amiodarone, atorvastatin, diltiazem and sildenafil. Grape fruit is a type of citrus fruit which has been widely used as a part of dieting regime and contains naringin, bergamottin, and 6,7-dihydroxybergamottin (DHB) which inhibit CYP3A4, a metabolizing enzyme for most drugs [47]. In this study, less than 50.00% of participants correctly knew the interactions between amiodarone, atorvastatin, sildenafil, diltiazem and grape fruit juice. The low knowledge found in this study is a cause of worry as the projected interaction risk of significant side effects for these drugs is assessed as high, with atorvastatin risking rhabdomyolysis and amiodarone risking Torsade de Pointes (TdP) [5].

On knowledge of drug-to-food time interval, the participants in this study exhibited an overall low knowledge of 32.55% which is similar to the study in South Africa (35.60%) [4] but lower than studies in Palestine and Jordan [6,19]. This therefore indicates that HCPs in this study have unsatisfactory knowledge of medications’ timing to food intake. Most of the HCPs were aware of the intake time of only omeprazole (58.50%), which is in line with previous studies that demonstrated adequate knowledge of the omeprazole food-time interval among HCPs [4,6,19,21,41]. Omeprazole should be taken 30 minutes before food for maximal activity [48]. The high knowledge score for omeprazole could be attributed to it being a proton pump inhibitor, which is the most commonly prescribed medications for gastroesophageal reflux disease, stomach ulcers and duodenal ulcers worldwide [49]. There was, however, low knowledge regarding the timing of drugs such as glipizide, isoniazid, griseofulvin, angiotensin-converting enzyme (ACE) inhibitors, propranolol, and NSAIDs with food in this study. This is quite similar to earlier studies reported among HCPs in different settings [3,4,6]. Drug administration before, with, or after food can influence drug absorption, distribution, metabolism, and excretion. For instance, while propranolol, ACE inhibitors and glipizide are well absorbed when taken on an empty stomach, the bioavailability of ketoconazole and albendazole are increased when taken with high-fat meal [3,6]. Hence, knowing the timing of medication administration relative to meals helps mitigate potential interactions that could affect drug absorption or bioavailability, thereby enhancing patient safety and treatment outcomes [50].

Regarding ADIs, the participants demonstrated moderately high knowledge (77.43%). The majority of the participants identified the interaction between alcohol and drugs like metronidazole, metformin, diazepam and co-trimoxazole. Comparable findings were also reported among pharmacists in Jordan [19]. Interactions between alcohol and drugs like metronidazole, sulfonylureas, or isoniazid can lead to disulfiram-like reactions characterized by flushing, headache, nausea, vomiting, sweating, and increased thirst [51]. Also, using alcohol concurrently with methotrexate, isoniazid, and paracetamol enhances the hepatotoxic effect of these medications [52]. Intake of antihistamines with alcohol may intensify their sedative effect, which can lead to accidents, falls, and injury and increase the risk of somnolence, ataxia, and respiratory depression [6,53,54]. Hence, HCPs need to be knowledgeable about these occurrences to prevent adverse outcomes for patients.

Overall, pharmacists had the highest knowledge score in all sections compared to the other HCPs. Similar findings have been reported in other studies [3,4,23,55]. In this study, HCPs’ knowledge of FDIs was not significantly associated with age or years of work experience, which is similar to other findings [4,19]. Some studies have, however, shown that work experience and age are strongly correlated with a medical professional’s knowledge of FDIs [6,21,41]. This study revealed that knowledge of HCPs’ on FDIs was associated with profession, attendance at an FDI training, and level of education. The multiple logistic regression model for overall knowledge on FDIs revealed that dietitians, medical doctors, midwives, and nurses are significantly less likely to have high overall knowledge in this area compared to pharmacists. This aligns with the findings reported in other studies [3,4]. The very small sample size of pharmacists may have had a bearing on the findings; hence, the results should be interpreted with caution.

The disparity in level of knowledge highlights the distinct educational and professional training that pharmacists receive in pharmacology and pharmacotherapy which inherently includes a deep focus on FDIs. Their education is specifically tailored to understanding drug interactions and their implications, providing pharmacists with specialized knowledge that other HCPs may not acquire to the same extent [56,57]. Furthermore, pharmacists are directly responsible for managing medications, including identifying and preventing adverse FDIs. Pharmacists frequently provide counselling to patients about how to take their medications, including instructions on food intake, which further solidifies their expertise [58–60]. These responsibilities reinforce their extensive knowledge of FDIs.

To help improve knowledge of HCPs on FDIs, it is essential to incorporate FDI education more thoroughly into the training curricula of medical, nursing, dietetic, and midwifery programs to ensure that all future HCPs develop a foundational understanding of FDIs. Furthermore, the knowledge disparity underscores the importance of interprofessional collaboration in healthcare settings. Pharmacists, with their specialized knowledge of FDIs, should be integral members of the healthcare team, providing critical insights that can prevent adverse interactions and improve patient outcomes [61]. Collaborative practice models where pharmacists work closely with medical doctors, nurses, dietitians, and midwives can enhance overall patient care [62]. Pharmacists can also play a pivotal role in educating other HCPs about FDIs. This was evident in an Iranian study, where the rate of FDIs was reduced in a hospital when a clinical pharmacist trained nurses about FDIs [63].

The data indicate that HCPs who have attended FDI training report higher knowledge scores compared to those who have not. Thus, there is a clear need for targeted continuing professional development (CPD) programs that address FDIs for all HCPs. Attendance at FDI training sessions is a form of CPD that keeps HCPs updated on the latest research and best practices. Regular participation in CPD activities has been shown to improve knowledge, skills, and attitudes, which in turn can lead to better patient care outcomes [64,65]. Therefore, healthcare institutions should consider making FDI training mandatory for all relevant staff. This could ensure that all HCPs have the necessary knowledge to identify and manage FDIs, thereby improving overall patient care. Furthermore, implementing interprofessional education programs can foster better collaboration and understanding among HCPs [66,67]. Collaborative interactions exhibit a blending of professional cultures and are achieved though sharing skills and knowledge to improve the quality of patient care [68,69]. Thus, by learning and practicing together, HCPs can develop a more comprehensive approach to managing FDIs, improving overall patient care. The majority of participants suggested in-service training, public education, CPD programs, and the active participation of pharmacists in ward rounds and clinical duties as means of improving knowledge of FDIs. In addition to trainings and CPD programs, structural and healthcare system barriers, such as limited access to resources, high patient workloads, and communication challenges between HCP groups may contribute to lower FDI knowledge among non-pharmacist HCPs. These factors can hinder ongoing learning and the practical application of FDI knowledge, exacerbating knowledge gaps. Addressing these barriers is essential for improving FDI knowledge and ensuring HCPs can effectively manage FDIs.

Another finding was the fact that HCPs with Bachelor’s/Master’s education reported higher knowledge score of FDIs than Certificate/Diploma holders indicating postgraduate degrees potentially expose you to advanced knowledge, hence, advocating for continued postgraduate education and training among HCPs [4,23].

The HCPs in this study demonstrated a moderately positive attitude towards FDIs, as almost half of them read prescription labels, interactions, precautions and directions in drug inserts before administering these medications to patients. The data highlighted significant differences in attitude scores among HCPs, with medical doctors reporting lower attitude scores compared to pharmacists, nurses, dietitians, and midwives. This discrepancy may be influenced by the fact that medical doctors often have extensive responsibilities that can lead to high levels of stress and burnout, which might negatively impact their attitudes towards certain critical aspects of patient safety as discovered in this study [70].

Furthermore, the analysis of the data revealed notable differences in attitude scores across various demographic groups. For instance, this study revealed that females have better attitude scores than males, indicating that gender can significantly influence attitudes and behaviors in healthcare settings. This gender difference could be attributed to variations in socialization processes, where females are typically more health-conscious and empathetic [71,72]. In addition, participants who attended FDI training workshops had significantly better attitude scores than those who did not. This finding underscores the critical role of educational interventions in shaping attitudes towards health practices. FDI education likely provides valuable knowledge and skills that empower individuals to make informed decisions about food and medication interactions. The findings from this study have significant implications for designing effective educational and other intervention programs. Firstly, gender-specific strategies may be necessary to address the attitude gaps between males and females. Secondly, promoting and facilitating attendance at FDI educational sessions could be a crucial strategy in improving attitudes toward FDIs, emphasizing the importance of continuous professional development and practical education.

The findings also indicate that HCPs who identify as Christians are more likely to exhibit positive attitudes towards FDIs than their non-Christian counterparts, highlighting the positive impact of religious beliefs on professional practice. This should however be viewed with caution as the majority of the participants were Christians. Religious beliefs often emphasize values such as compassion, empathy, and holistic care, which are fundamental in healthcare practice, and can influence health behaviors and attitudes towards medical practices [73,74]. This perspective may lead to a more thorough consideration of factors affecting health, including FDIs. Healthcare institutions could therefore develop tailored educational programs that consider the religious backgrounds of their HCPs.

The premise that HCPs with high knowledge of FDIs will exhibit a higher attitude if they also possess high knowledge of ADIs suggests a potential synergy between different aspects of pharmacological and pharmacotherapeutic knowledge and their impact on professional attitudes. HCPs who demonstrate high knowledge in both FDIs and ADIs are likely to exhibit greater confidence in their ability to manage complex medication regimens. This confidence can positively influence their attitudes towards incorporating evidence-based practices into clinical decision-making processes.

In addition, participants with high knowledge of drug-to-food time intervals will exhibit better attitudes, and these attitudes can be further improved with high knowledge of ADIs. Knowledge of drug-to-food time intervals is crucial for HCPs to ensure optimal medication absorption and effectiveness [50]. Knowledge of ADIs enables HCPs to identify and manage potential risks associated with alcohol consumption and medication use. This includes understanding how alcohol can potentiate or diminish the effects of certain medications, leading to informed counselling and guidance for patients on safe alcohol consumption practices [75,76]. Thus, HCPs who possess comprehensive knowledge of both drug-to-food time intervals and ADIs are likely to exhibit enhanced attitudes towards safe medication practices. This dual proficiency enables professionals to adopt a proactive approach to managing complex medication regimens, leading to improved patient safety and satisfaction.

## Limitations and strengths

Despite potential limitations such as self-selection bias and the reliance on self-reported knowledge assessments, several factors strengthen the validity and applicability of our findings regarding FDIs among HCPs at HTH. Our study achieved a high response rate among eligible HCPs at HTH, minimizing the potential impact of self-selection bias. In addition, the demographic characteristics of our respondents closely resemble the overall HCP population at the hospital in terms of age distribution, professional categories, and years of experience, suggesting our sample is representative of the target population. Data was also collected during different shifts and across various departments, ensuring broad participation and reducing systematic exclusion of specific healthcare provider groups. Concerning self-reported knowledge assessment, the study employed validated assessment tools that have been previously used in similar settings, enhancing the reliability of self-reported measures. Furthermore, the anonymity of responses likely encouraged honest self-assessment, reducing social desirability bias in knowledge reporting.

Generalizability of the findings is also a crucial consideration. While the study may offer valuable insights into FDIs in the specific context of HCPs in Ghana, the findings may not be applicable to other regions or populations. Therefore, a more comprehensive nationwide, or multi-center study with a larger sample size is suggested for a more generalized conclusion. The results of the dietitians and pharmacists need to be interpreted with caution, considering the very small sample, compared to the medical doctors, nurses and midwives.

Regardless of these limitations, our findings provide valuable baseline data for future interventions and policy development. The results align with similar studies conducted in comparable settings, suggesting the identified patterns reflect genuine trends in HCP knowledge and attitudes regarding FDIs.

## Conclusion

The findings of this study indicate that knowledge of FDIs among HCPs is inadequate. The HCPs had very high general knowledge of FDIs and ADIs but low knowledge of the specific foods that can interact with drugs. In addition, the correct timing of food intake relative to specific drugs was low. The study revealed that knowledge of HCPs’ on FDIs was associated with profession, attendance at FDI training, and level of education. Overall, pharmacists had the highest knowledge score in all sections compared to the other HCPs. The HCPs in this study showed a moderately positive attitude toward FDIs, with pharmacists showing the highest. HCPs need to be conversant with common FDIs to provide appropriate patient counselling and achieve better therapeutic outcomes. This study, therefore, emphasizes the need for educational programs to increase understanding about FDIs among HCPs.

## Recommendations

There is a need to emphasize continuing education or incorporating detailed coverage of FDIs in the curricula of HCPs, focusing particularly on those areas identified as gaps in knowledge. Furthermore, the development of standardized training programs or tools aimed at improving the understanding of FDIs should be considered. These training programs could be made part of the pre-registration training or as part of CPD to ensure an updated understanding of this demanding healthcare practice. Posters and banners of significant FDIs can be posted on the walls of health facilities to constantly stimulate HCPs and increase their knowledge. Furthermore, implementing integrated care models where pharmacists are actively involved in patient care teams can ensure that the expertise regarding FDIs is utilized effectively.

## Future research directions

Further research is needed to explore the specific elements of pharmacy education that contribute to higher FDI knowledge and how these can be integrated into other healthcare curricula. For instance, conducting research to assess the impact of structured FDI training programs integrated into the curricula of various healthcare disciplines would be highly valuable. Furthermore, investigating comparative studies to determine the most effective educational strategies across different healthcare professions—such as workshops, interactive modules, or case-based learning—could provide important insights. There is also the need to explore the underlying causes of the attitudinal differences observed. Longitudinal studies could provide insights into how attitudes evolve with different professional experiences and educational interventions. Additionally, qualitative research methods, such as in-depth interviews and focus groups, could offer a deeper understanding of the personal and professional experiences that shape these attitudes.

## Supporting information

S1 File R Codes for comparing knowledge scores by groups. (DOCX)

S2 File Questionnaire. (DOCX)

S3 File The data used for Modelling Knowledge and Attitude Towards Food-Drug Interactions among Healthcare Professionals. (XLSX)
