# Supplementary material for: Food-drug interactions: Modelling knowledge and attitude among healthcare professionals at the Ho Teaching Hospital
Source: PLoS One. 2025 May 22;20(5):e0323793. doi: 10.1371/journal.pone.0323793 (PMC12097585; doi:10.1371/journal.pone.0323793)
Supplement: S1 File — (DOCX) [file pone.0323793.s001.docx]

**Codes for comparing knowledge scores by groups**

kruskal.test(ATT~Year_of_experience,

data = Data)

library(FSA)# For TIES

dunnTest(ATT~Year_of_experience,

data = Data,

method = "bh"

)

kruskal.test(KG2~AgeCode,

data = Data)

dunnTest(KGT~Profession,

data = Data,

method = "bh"

)

kruskal.test(KG3~Year_of_experience,

data = Data)

dunnTest(KG3~Year_of_experience,

data = Data,

method = "bh"

)

kruskal.test(KG4~Year_of_experience,

data = Data)

dunnTest(KG4~Year_of_experience,

data = Data,

method = "bh")

kruskal.test(KGT~AgeCode,

data = Data)

dunnTest(KGT~Year_of_experience,

data = Data,

method = "bh")

wilcox.test(ATT ~ FDI_attendance, data = Data, exact = FALSE)

wilcox.test(KG1 ~ FDI_attendance, data = Data, exact = FALSE)

wilcox.test(KG2 ~ Religion, data = Data, exact = FALSE)

wilcox.test(KG3 ~ FDI_attendance, data = Data, exact = FALSE)

wilcox.test(KG4 ~ FDI_attendance, data = Data, exact = FALSE)

wilcox.test(KGT ~ Education_level, data = Data, exact = FALSE)

**Codes for logistic regression models**

M1 = glm(KGTC~Sex+Religion+Profession+Education_level+Year_of_experience+

FDI_attendance+AgeCode,data = Data,

family = binomial(link = logit))

library(rcompanion)

compareGLM(M1)

summary(M1)

M2 = glm(ATTC~Sex+Religion+Profession+Education_level+Year_of_experience+

FDI_attendance+Sex:AgeCode,data = Data,

family = binomial(link = logit))

compareGLM(M2)

summary(M2)

**Codes for checking assumptions and Mediation analysis**

library(lavaan)

myData<-data.frame(ATT,KG4,KG2,KG3)

cor(myData)

library(REdaS);bart_spher(myData, use = "pairwise.complete.obs")

KMOS(myData, use = "pairwise.complete.obs")## sampling adequacy

library(MVN);mvn(myData, subset = NULL, mvnTest = c("mardia"), multivariatePlot = "qq")

model1<-'

ATT~a*KG4+b*KG2

KG4~c*KG2

Indirect1:=a*c '

Model2<-'

ATT~a*KG4+b*KG3

KG4~c*KG3

Indirect1:=a*c '

fit1<-sem(model,data=Data,estimator = "ML")

summary(fit1, fit.measures=TRUE)
