# Supplementary material for: Food-drug interactions: Modelling knowledge and attitude among healthcare professionals at the Ho Teaching Hospital
Source: PLoS One. 2025 May 22;20(5):e0323793. doi: 10.1371/journal.pone.0323793 (PMC12097585; doi:10.1371/journal.pone.0323793)
Supplement: S2 File — (DOCX) [file pone.0323793.s002.docx]

**QUESTIONNAIRE**

KNOWLEDGE AND ATTITUDE TOWARDS FOOD-DRUG INTERACTIONS AMONG HEALTHCARE PROFESSIONALS AT THE HO TEACHING HOSPITAL.

RESPONDENT’S CODE …………….. DATE …………………

**SECTION A: SOCIODEMOGRAPHIC CHARACTERISTICS**

**(Please select one answer by ticking or writing your answer in the blank spaces provided)**

Q1. Age (years) ………

Q2. Sex


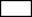
 Male
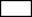
 Female

Q3. Religion


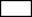
 Christianity
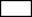
 Islamic
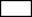
 Traditionalist Others…………………..

Q4. Profession


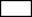
 Doctor
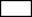
 Pharmacist
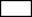
 Nurse
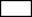
 Dietitian
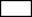
 Midwife

Q5. Level of education


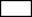
 Diploma
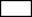
 Bachelor’s degree
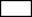
 Master’s degree
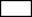
 PhD others (please specify)…………………………

Q6. Year of experience


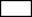
 0-4 yrs
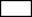
 5-9 yrs
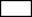
 10-14 yrs
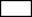
 15-19 yrs
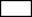
 >20 yrs

Q7. Have you attended any training where you were informed about food-drug interactions?


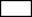
 Yes
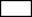
 No
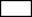
 I don’t know

**SECTION B: LEVEL OF KNOWLEDGE ON INTERACTIONS BETWEEN FOODS AND SOME SPECIFIC DRUGS**

|  | Questions 1- 4, Please tick either Yes or No | YES | NO |
| --- | --- | --- | --- |
| Q1. | Some foods can interfere with the effectiveness of drugs in the body? |  |  |
| Q2. | Some drinks can interfere with the effectiveness of drugs in the body? |  |  |
| Q3. | Some foods can increase or decrease the action of a drug? |  |  |
| Q4. | Some drugs can alter the nutritional status of a patient? |  |  |

**Please TICK all the applicable answers for the questions below**

Q5. Patients taking **theophylline** should avoid consuming large quantities of……..


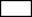
 Tea
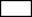
 Coffee
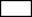
 chocolate

Q6. Patients on **diazepam** should avoid consuming…


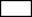
 Tea
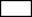
 Coffee
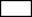
 Cola
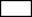
 Energy drinks

Q7. Patients taking antibiotics like **tetracycline** and **fluoroquinolone** should avoid taking


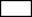
 Milk
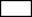
 Dairy products
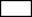
 Meat
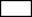
 Iron rich food

Q8. A patient taking **antibiotics** should avoid acidic foods such as


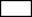
 Tomato sauce
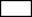
 Potato
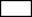
 Citrus juice
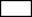
 Coffee

Q9. Patients on **warfarin** should avoid foods like ……..


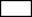
 Spinach
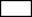
 Broccoli
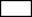
 Green leafy vegetables
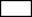
 Pork
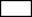
 mushroom

Q10. Patients on **spironolactone** should avoid


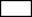
 Spinach
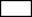
 Broccoli
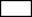
 Green leafy vegetables

Q11. Patients on **MAOIs** like **phenelzine, selegiline** and **tranylcypromine** should avoid …..


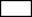
 Cheese
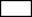
 Wine
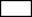
 Beer
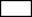
 Fermented foods

Q12. Can **amiodarone** be taken with **grapefruit juice?**


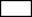
 Yes
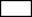
 No
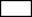
 I don’t know

Q13. Can **atorvastatin** be taken with **grapefruit juice**?


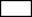
 Yes
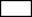
 No
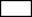
 I don’t know

Q14. Can **Diltiazem** be taken with **grapefruit juice**?


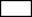
 Yes
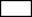
 No
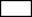
 I don’t know

Q15. Can **Sildenafil** (Viagra) be taken with grapefruit juice?


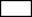
 Yes
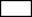
 No
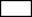
 I don’t know

Q16. Patients taking **levodopa** should avoid foods rich in protein like……..


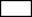
 Beef
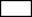
 Chicken
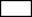
 Pork
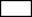
 Egg

Q17. Patients on **levothyroxine** should avoid foods like ………


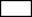
 Cabbage
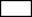
 Millet
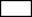
 Lean meat
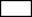
 Cauliflower

**SECTION C: KNOWLEDGE OF DRUG-TO-FOOD-TIME INTERVAL**

**(Please select the appropriate answer by ticking the box)**

Q18. **Zidovudine** can be taken without relation to food intake


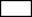
 Yes
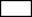
 No
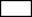
 I don’t know

Q19. **Didanosine** and **Indinavir** must be taken on empty stomach


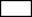
 Yes
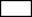
 No
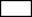
 I don’t know

Q20. **Lopinavir** / **Ritonavir** must be taken ……..


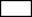
 with food
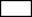
 before food
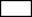
 after food
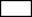
 I don’t know

Q21. **Propranolol** must be taken on empty stomach


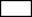
 Yes
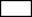
 No
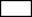
 I don’t know

Q22. **ACE inhibitors** like **Lisinopril** should be taken on empty stomach


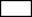
 Yes
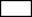
 No
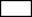
 I don’t know

Q23. **Esomeprazole/ omeprazole** should be taken


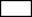
 with food
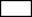
 before food
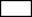
 after food
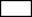
 I don’t know

Q24. **Griseofulvin** and **Albendazole** should be taken with high fat foods


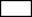
 Yes
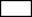
 No
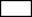
 I don’t know

Q25. **Metformin** should be taken …….


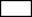
 with food
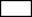
 before food
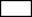
 after food
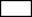
 I don’t know

Q26. **Glipizide** should be taken ……..


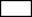
 with food
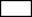
 before food
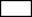
 after food
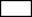
 I don’t know

Q27. **Isoniazid** should be taken……

with food before food after food I don’t know

Q28. **NSAIDs** and **steroids** should be taken

with food before food after food I don’t know

**SECTION C: KNOWLEDGE ON ALCOHOL-DRUG INTERACTIONS**

**(Please select the appropriate answer by ticking the box)**

Q29. Patients on **metronidazole** should avoid alcohol intake

Yes No I don’t know

Q30. Patients on **antihistamine** like **cimetidine** should avoid alcohol intake

Yes No I don’t know

Q31. Patients on **metformin** should avoid alcohol intake

Yes No I don’t know

Q32. **Isoniazid** should not be taken with alcohol

Yes No I don’t know

Q33. Patients on **diazepam** should avoid alcohol intake

Yes No I don’t know

Q34. Patients on **methotrexate** should avoid alcohol intake

Yes No I don’t know

Q35. Patients on sulfonamides like **co-trimoxazole** should avoid alcohol intake

Yes No I don’t know

**SECTION D: ATTITUDE TOWARDS FOOD-DRUG INTERACTIONS**

**(Please select the appropriate answer by ticking the box)**

Q36. How often do you read prescription label before administering/giving out drug to patients?

Always Sometimes Not at all

Q37. How often do you read directions, interactions, precautions on drug inserts before administering it?

Always Sometimes Not at all

Q38. How often do you counsel patients on potential food-drug interaction before dispensing the drugs?

Always Sometimes Not at all

Q39. Have you reported any food-drug interaction to authorities before?

Yes No Not sure

Q40. Has a patient ever reported food drug interaction to you before?

Yes No Not sure

Q41. How do you think knowledge of food drug interactions can be improved?

Please give your suggestions…………………………………………………..
